# Genetic Transformation System for Woody Plant *Tripterygium wilfordii* and Its Application to Product Natural Celastrol

**DOI:** 10.3389/fpls.2017.02221

**Published:** 2018-01-09

**Authors:** Yujun Zhao, Yifeng Zhang, Ping Su, Jian Yang, Luqi Huang, Wei Gao

**Affiliations:** ^1^State Key Laboratory Breeding Base of Dao-di Herbs, National Resource Center for Chinese Materia Medica, China Academy of Chinese Medical Sciences, Beijing, China; ^2^School of Traditional Chinese Medicine, Capital Medical University, Beijing, China

**Keywords:** *Tripterygium wilfordii*, genetic transformation, particle bombardment, suspension cell, celastrol

## Abstract

*Tripterygium wilfordii* is a perennial woody liana medicinal plant with several crucial biological activities. Although studies on tissue culture have previously been conducted, research on genetic transformation is much more challenging and therefore results in slower progress. In the present study, a highly efficient transformation system involving the particle bombardment of *T. wilfordii* with the reporter *egfp* gene using the PDS-1000/He system was established. A total of seven parameters affecting the genetic transformation were investigated using an L_18_ (6 × 3^6^)-type orthogonal array. The result indicated that DNA delivery conditions of 3-cm target distance, 1100 psi helium pressure, 28 mmHg chamber vacuum pressure, three times number of bombardment, CaCl_2_ as precipitation agent, 2 μg plasmid DNA concentration and 48 h post-bombardment incubation time were optimal for *T. wilfordii* cell suspensions transformation. The average transformation efficiency was 19.17%. Based on this transformation system, the overexpression of two *T. wilfordii* farnesyl pyrophosphate synthase genes (*TwFPSs*) was performed in cell suspensions. Integration of the *TwFPSs* in the genome was verified by PCR analysis and also by Southern blotting using hygromycin gene as a probe. Real-time quantitative PCR analysis showed that the expression of *TwFPS1&2* was highly up regulated in transgenic cell suspensions compared with control cells. The detection of metabolites showed that *TwFPS1*&*2* could highly increase the celastrol content (973.60 μg/g) in transgenic cells. These results indicated that this transformation system is an effective protocol for characterizing the function of genes in the terpenoid biosynthetic pathway.

## Introduction

*Triptergium wilfordii* Hook. F., also known as *Gelsemium elegans* or herba fibraureae recisae, is a perennial woody liana medicinal material found in China, Korea and Japan. As a Celastraceae plant, *T. wilfordii* shows obviously effects on immune regulation and exhibits anti-inflammatory, anti-tumor, anti-fertility and antimicrobial activities. Clinically, *T. wilfordii* has been used to treat autoimmune diseases (Zhou et al., 2012; Venkatesha and Moudgil, 2016) such as rheumatoid arthritis (Landewé and van der Heijde, 2014), systemic lupus erythaematosus (SLE) (Vagnani et al., 2015) and nephritis (Zhu et al., 2013). The chemical components of *T. wilfordii* are complicated, with effective constituents including alkaloid, diterpenes, triterpenes and sesquiterpenoids (Liu et al., 2011). Among these compounds, the triterpene celastrol was initially extracted from the roots in Chou and Mei (1936). Yang et al. (2006) showed that celastrol is an effective natural protease inhibitor that induces prostate cancer cell apoptosis (Li-Weber, 2013). A series of subsequent studies showed that celastrol exhibits pharmaceutical activities, such as anti-tumor, anti-rheumatism, analgesia activities (Ríos, 2010; Kannaiyan et al., 2011; Wang et al., 2016). Moreover, there have been reported that celastrol is a leptin sensitizer and a promising agent for the pharmacological treatment of obesity (Greenhill, 2015; Liu et al., 2015; Ma et al., 2015).

However, the content of these secondary metabolites was low in *T. wilfordii*, and wild resources have gradually been reduced, as some plants have faced extinction. The roots of *T. wilfordii* is the medicinal portion used in Chinese medicine and these roots could be harvested at a diameter of 2–3 cm 7 years after planting. But the chemical extraction of effective components is difficult to meet demands. Synthetic biology in the past several years has rapidly developed (Breitling et al., 2013). Indeed, synthetic biology has been designed, regulated and optimized on different levels to produce new drugs, biofuels, and target products to maximize production (Gechev et al., 2014). In recent years, terpenoid biosynthetic pathway analysis has been the most important part of the research in *T. wilfordii* (Li et al., 2009; Wu et al., 2012; Liu et al., 2014; Zhang et al., 2015; Zhao et al., 2015). Our ongoing studies were focus on identifying preciously uncharacterized related genes in terpenoids metabolism of *T. wilfordii*. To identify these unknown genes in *T. wilfordii* cell suspensions and to rabidly characterize potential genes that can be used in the biosynthesis of targeted production, a suitable transformation system for the transgenes into *T. wilfordii* cell suspensions must be established.

Genetic transformation of *T. wilfordii* is poorly advanced compared to other medicinal plants (Kikkert et al., 2005; Zárate and Verpoorte, 2007; Nehra et al., 2010; Purkayastha et al., 2010). Woody plant is different from herbaceous, its plants or explants grow slowly (Kramer, 2012), and establishing stable expression patterns requires more time (Birch, 1997). *Agrobacterium*-mediated transformation and particle bombardment-mediated are the two widely used methods for plant genetic transformation (He et al., 2010). *Agrobacterium*-mediated transformation studies on *T. wilfordii* have been demonstrated on hairy root culture (Nakano et al., 1998; Zhu et al., 2014). Particle bombardment-mediated transfromation is a physical process, and it offers no biological constraints and host limitations (Altpeter et al., 2005). In the previously, numerous woody plant transformation studies were reported by particle bombardment-mediated transformation system(Christou, 1995; Cem et al., 2003; Clapham et al., 2003; Malabadi and Nataraja, 2007) and the highest transformation efficiency was 13% (Song et al., 2006). Low efficiency of genetic transformation often results from many factors, such as materials (Plackett et al., 2014; Zhang et al., 2014; Yadava et al., 2017) and bombardment parameters. Therefore, selecting appropriate transgenic expression vectors and organization and optimizing the parameters to improve transformation efficiency are required (Ahmad et al., 2017).

The aim of this paper was to develop approaches allowing stable transformation on cell suspensions, especially for gene function studies. Farnesyl pyrophosphate synthase (FPS) catalyzes the head-to-tail condensation reaction of dimethylallyl pyrophosphate (DMAPP) with two molecules of isopentenyl pyrophosphate (IPP) to form farnesyl pyrophosphate (FPP) (And and Poulter, 2006). FPP is the precursor of all sesquiterpenes, such as artemisinin or wilfordine (Zhao et al., 2015). Daudonnet transformed yeast *FPS* into tobacco and observed that the sterol and carotenoid content in transgenic plants significantly increased (Daudonnet et al., 1997). The cotton *FPS* was transformed into *Artemisia annua*, and the artemisinin content in 5 transgenic lines was approximately 2- to 3-fold higher that than in control plants, reaching 8–10 mg⋅g-1 DW (Chen et al., 2000). The overexpression of *FPS1* in *Artemisia annua* resulted in 2–3 times higher *FPS* expression than in untransformed *Artemisia annua*, and artemisinin expression was approximately 34.4% higher than that in untransformed *Artemisia annua* (Han et al., 2006). Tobacco plants transformed with *Mentha arvensis FPS* were strongly antagonistic to brown spot disease (Hong et al., 2006; Cui et al., 2007). The overexpression of *PgFPS* in *Centella asiatica* increased the expression of CADDS and *CaCYS* in hairy roots, revealing that *FPS* could change the activity of triterpenoid biosynthesis (Kim et al., 2010). All these previous studies have indicated that *FPS* could increase the isoprenoid content in transformed plants. Applied to *TwFPS* functioning, this paper studied the use of particle bombardment-mediated transformation on cell suspensions, as a convenient DNA delivery technique to induce stable gene expression.

## Materials and Methods

### Plasmid DNA

The PBI-1300-EGFP (**Supplementary Figure S3**, supplied by Pro. Meng Wang, Institute of Genetics and Developmental Biology Chinese Academy of Sciences) plasmid carries *egfp* reporter gene and a selective marker gene hygromycin, driven by a CaMV 35S promoter. The vector was used to transform the cells to optimize the factors influencing particle bombardment. Vector pH7WG2D (Saved by our laboratory) was used to overexpress genes *TwFPS1*&*2* in cells. The plasmid DNA was extracted using Plasmid Maxi Kit (Omega, United States).

### Construction of pH7-*TwFPS1* and pH7-*TwFPS2*

The full length of *TwFPS1* and *TwFPS2* was obtained in the previous experiments (Zhao et al., 2015). Design the primers FPS1-attF/R and FPS2-attF/R (**Supplementary Table S1**) and use super fidelity enzyme Phusion High-Fidelity PCR Kit (NEB, United States) for PCR amplification.

We used Gateway procedure to construct the two genes into the entry vector pENTR/SD/D-TOPO (**Supplementary Figure S4**, Invitrogen, United States) through BP reaction. As the Gateway Technology described, add components in a 200 μl microcentrifuge tube as tabulated below:

**Table:** 

**Component**	**Amount (μl)**

Fresh PCR product	0.5
Salt Solution	2
TOPO vector	0.5
Final volume	3


Mix well by vortexing and incubate at 25°C for 1 h. Then add 0.5 μl Proteinase K solution and incubate at 37°C for 10 min. Transform competent *Escherichia coli (E. coli)* and select for the kana (50 mg/L) antibiotic-resistant entry clones. The plasmid was detected by PCR using primers M13F/R. Ligating the fragment to vector pH7WG2D (**Supplementary Figure S5**) by LR reaction. As the Gateway Technology described, add components in a 200 μl microcentrifuge tube as tabulated below:

**Table:** 

**Component**	**Amount (μl)**

Entry clone (100–300ng)	0.4
Destination vector (150ng)	1
5 × LR Clonase reaction buffer	1
TE Buffer, pH 8.0	0.6
Final volume	3


Mix well by vortexing and incubate at 25°C for 3 h. Then add 0.5 μl Proteinase K solution and incubate at 37°C for 10 min. Transform competent *E. coli* and select for the spectinomycin (100 mg/L) antibiotic-resistant entry clones. The plasmid was detected by PCR using primers FPS1/2-attR and PH7-F (**Supplementary Table S1**). Finally the plasmid was sequenced to verify if vector construction successfully.

### Cell Suspensions Culture and Multiplication

Fresh leaves of *T. wilfordii* were sheared, cleaned and disinfected. Then, the leaves were cut into 1.0 cm × 1.0 cm pieces after rinsing with sterile water. These leaves were cultivated in Murashige & Skoog (MS, PhytoTechnology, United States) agar medium containing 1.0 mg/L 2,4-Dichlorophenoxyacetic acid (2,4-D) at 25°C in dark. After 2 weeks, calluses began to grow at the explant slits. The calluses which had a white luster, were soft and grew well, were cultured in MS agar medium containing 0.5 mg/L of 2,4-D, 0.1 mg/L of Kinetin (KT), and 0.5 mg/L of Indole-3-butyric acid (IBA) at 25°C in the dark.

After 3 subcultures, we chose the calluses that grew well and that had a loose texture and clipped these calluses into small pieces with tweezers. These calluses were cultured in MS medium containing 0.5 mg/L of 2,4-D, 0.1 mg/L of KT, 0.5 mg/L of IBA, and cell suspensions of 2.0 g/40 mL in the dark at 25°C with rotary shaking at 120 rpm (Zhao et al., 2015).

### Screening of Hygromycin-Resistant Transformants

Hygromycin selection marker was combined with *egfp* for the selection of putative transformed cells. The antibiotic was added to selection MS liquid medium supplemented with 3% sucrose, 0.5 mg/L 2,4-D, 0.1 mg/L KT, and 0.5 mg/L IBA, pH 5.8. A range of hygromycin concentrations (0, 0.1, 0.5, 1, 1.5, 2, 2.5, 3, 3.5, 5, 10, 20, 30, 40, 50, 60, 70, 80, 90, and 100 mg/L) was examined to screen the critical concentration. Each treatment was repeated three times, and then suction filtration was performed, and the cell suspensions at logarithmic growth phase were weighed to determine the critical concentration.

### Orthogonal Design of Experiments

According to previous literature (Rasmussen et al., 1994; Hilliou et al., 1999; Rajasekaran et al., 2000; Chong and Maziah, 2005; Vongpaseuth et al., 2007; Guirimand et al., 2009; Xun et al., 2011; Jagga-Chugh et al., 2012; Rajasekaran, 2013; Mousavi et al., 2014), the optimization parameters included target tissue distance, helium pressure, chamber vacuum pressure, number of bombardments, precipitation agents, plasmid DNA concentration and post-bombardment incubation time. These parameters and the levels of the variables studied are reported in **Table 1**.

**Table 1:** Control factors and levels of variables used in this study.

Symbol	Control factor	Levels					
		
		1	2	3	4	5	6
A	Target tissue distance (cm)	3	6	9	-	-	-
B	Helium pressure (psi)	900	1100	1350	-	-	-
C	Chamber vacuum pressure (mmHg)	26	27	28	-	-	-
D	Number of bombardments	1	2	3	-	-	-
E	Precipitation agents	C^a^	S^b^	CS^c^	-	-	-
F	Plasmid DNA (μg)	1	1.5	2	–	–	–
G	Time (hours)	24	48	72	96	120	144


*^a^Plasmid DNA was coated onto gold particles with the present of calcium chloride only. ^b^Plasmid DNA was coated onto gold particles with the present of spermidine only. ^c^Plasmid DNA was coated onto gold particles with the present of both calcium chloride and spermidine.*

Based on the above seven factors, an orthogonal array with mixed levels of L_18_ (6 × 3^6^) was used. **Table 2** shows the design of the orthogonal array. One six-level parameter (post-bombardment incubation time) and six three-level parameters (target tissue distance, helium pressure, chamber vacuum pressure, number of bombardments, precipitation agents, plasmid DNA concentration) were utilized. Each factor was repeated three times, and the entire experiment was repeated twice.

**Table 2:** The orthogonal array with mixed levels of L_18_ (6 × 3^6^) (numbers 1–6 show the different levels of the each factors as assigned in **Table 1**).

Run	Control factors						
	
	Time (hours)	Target tissue distance (cm)	Helium pressure (psi)	Number of bombardments	Precipitation agents	Plasmid DNA (μg)	Chamber vacuum pressure (mmHg)
1	1	1	3	2	2	1	2
2	1	2	1	1	1	2	1
3	1	3	2	3	3	3	3
4	2	1	2	1	2	3	1
5	2	2	3	3	1	1	3
6	2	3	1	2	3	2	2
7	3	1	1	3	1	3	2
8	3	2	2	2	3	1	1
9	3	3	3	1	2	2	3
10	4	1	1	1	3	1	3
11	4	2	2	3	2	2	2
12	4	3	3	2	1	3	1
13	5	1	3	3	3	2	1
14	5	2	1	2	2	3	3
15	5	3	2	1	1	1	2
16	6	1	2	2	1	2	3
17	6	2	3	1	3	3	2
18	6	3	1	3	2	1	1


### Optimized Protocol of Transformation of *T. wilfordii* Cell Suspensions

Logarithmic growth cells was vacuum filtered on a 35-mm diameter petri dish and plated on MS solid medium supplemented with 3% sucrose, 0.5 mg/L 2,4-D, 0.1 mg/L KT, 0.5 mg/L IBA, pH 5.8. Preculture for 7 days in case of bombardment.

Approximately 30 mg of Au microparticles in 1 ml of 70% ethanol (v/v) was added, followed by vortexing for 3–5 min. The mixture was left standing for 15 min at room temperature, followed by centrifugation for 5 min at 10,000 rpm. Subsequently, the pellet was resuspended in 1 ml of sterile water for 1 min, with standing for 1 min. Following centrifugation, the supernatant was removed, and the cells were cleaned twice as described above. Finally, 500 μl of 50% sterile glycerol was added, followed by intensive mixing. The microparticles (60 mg/ml final concentration) can be stored for 2 weeks at room temperature.

An aliquot of 50 μl of the microparticles was mixed with 5 μl of DNA (concentration according to **Table 2**), and 50 μl of 2.5 M CaCl_2_ and 20 μl of 0.1 M spermidine were added to coat the microparticles after vortexing for 2–3 min, with standing for 1 min, followed by centrifugation for 2 s and the removal of the supernatant. The pellet was washed with 140 μl of 70% ethanol (v/v), followed by centrifugation and the removal of the supernatant. Subsequently, the sediment was washed with 100% ethanol (v/v), followed by centrifugation and removal of the supernatant. A total of 48 μl of 100% ethanol (v/v) was added, and the pellet was moderately resuspended for 2–3 s. Approximately 8–10 μl of the suspension was spread above the microcarrier fitted over the microcarrier holder. To avoid the agglomeration of the microcarrier, the suspension was resuspended 2–3 s prior to every bombardment.

Bombard the cell suspensions with biolistic gene gun (PDS 1000/He, Bio-Rad), choosing the plasmid DNA in according with **Table 2**. The several of combinations were listed on **Table 2**. Each run was carried out two times. Then depending on the requirements for incubation. Bombarded cells were observed for expression of *egfp* through Laser Scanning Confocal Microscope (LSCM). Cell suspensions were examined under a LSM880NLO microscope (Zeiss, German) using an excitation wavelength of 488 nm.

### Statistical Analysis of Orthogonal Experiment

The optimization experiment was repeated two times. The bombardment effect was evaluated by the number of *egfp* spots per square centimeter. All statistical analyses were performed at the level 5% using Microsoft Office Excel and SPSS 18.0 (SPSS Inc. United States).

The order and contribution rate of every experiment factor on *egfp* spots was determined by means of range analysis and variance analysis respectively. In range analysis, calculate the mean value of K_i_ and R. Among them:

$$R=\max\left({\overset{¯}{K}}_{1}\right)-\min\left({\overset{¯}{K}}_{1}\right)$$

As the mixed orthogonal experiment, adjust the R to R′:

$$R^{\prime }=dR\sqrt{r}$$

The influence of each factor on the target index could be determined according to the R. The larger the *R* value, the greater the influence of the factors on the target index. The specific calculation method was showed in **Supplementary Table S2**.

The range analysis is simple and obvious, but it is not possible to distinguish the data fluctuation caused by experimental conditions or caused by experimental error. Variance analysis can be used to make up for the defects of range analysis. Variance analysis resolved the total variation into factor variation and error variation. The significance of the parameter was determined by *F*-test. The specific calculation method was showed in **Supplementary Table S3**.

### Transformation Efficiency Analysis by Imaging Flow Cytometry

After optimization experiment we used the optimum condition to transform the cell suspensions. The bombarded cell suspensions were incubated for 48h on MS solid medium supplemented with 3% sucrose, 0.5 mg/L 2,4-D, 0.1 mg/L KT, 0.5 mg/L IBA, pH 5.8. Image flow cytometry was applied to examine the transformation efficiency of this method. After transformation, the nuclei were released as described (Xu et al., 2011). Briefly, the cell suspensions were chopped using a razor blade in ice-cold Galbraith’s buffer (45 mM MgCl_2_, 30 mM sodium citrate, 20 mM MOPS, 0.1% (w/v) TritonX-100, pH 7.0). Then filtering and DAPI (2 μg/mL) staining, the nuclei were analyzed by FlowSight imaging cytometer (Amnis Corp. Seattle WA, United States). The system was equipped with 405, 488 nm, side scatter (SSC) 785 nm excitation lasers. For acquisition, violet 405 nm, blue 488 nm, and 785 nm laser powers were set to 25, 60, and 7.5 mW respectively, channels 1 and 6 were set as the brightfield and SSC.

### Selection and Subculture of Transformed Cell Suspensions

On the basis of the combination of conditions, we transformed the two genes *TwFPS1* and *TwFPS2* into cell suspensions respectively. The transformed cell suspensions were incubated for 2 days on MS medium containing 0.5 mg/L 2,4-D, 0.1 mg/L KT, 0.5 mg/L IBA (pH 5.8) and then transferred onto selection medium (MS medium supplemented with 0.5 mg/L 2,4-D, 0.1 mg/L KT, 0.5 mg/L IBA) combined with 2.5 mg/L hygromycin for selection of transformed cell suspensions.

After 21 days of shaking culture, the positive cell suspensions could not be found. The transformed cell suspensions T1 were obtained after 2 generations of subculture for more than 40 days. These cell suspensions were collected and stored at -80°C. After several generations of screening and reducing the concentration of hygromycin antibiotics gradually, two generations (T2, T3) of transformed cell suspensions were harvested to detect the celastrol content.

### PCR and Southern Blot Analysis

PCR amplification and Southern blot analysis were carried out to verify the integration of the hygromycin gene into the genome. Total cellular DNA was extracted from transformed cell suspensions and wild-type (WT) cell suspensions based on the cetyltrimethyl ammonium bromide (CTAB) method (Murray and Thompson, 1980). Two pairs of primers PH7 EGFP F/R and Hm F/R were used to verified the integration of the *TwFPS* gene. Thirty-five PCR cycles were used for amplification (with denaturation at 94°C for 30 s, an annealing at 55°C for 30 s, and an elongation at 72°C of 1 min and further final extension at 72°C for 5 min), after an initial denaturation step at 94°C for 5 min. The PCR products were electrophoresed on a 1% agarose gel.

Southern blot hybridization was carried out with 10 μg of total cellular DNA digested with *SacI* (Takara). The digested DNA fragments were separated by electrophoresis at 25 V in a 0.7% agarose gel, before being transferred to a nylon membrane (Hybond N+, Amersham). A 512 bp PCR product of the part of Hm gene was used as a probe for Southern blot hybridization. The PCR product was purified using a PCR purification kit (Qiagen). The probe was labeled with dUTP using PCR DIG Probe Synthesis Kit (Roche) and hybridized overnight at 37°C with agitation in a hybridization oven. The membrane was washed with 2 × SSC/0.1% SDS and 1 × SSC/0.1% SDS at 65°C. an autoradiogram was obtained after 1h exposure with CSPD.

### qRT-PCR Analysis

Total RNA was extracted from cell suspensions with the Total RNA Kit (Promega). The primers (qRT-β-Actin, qRT-FPS1, qRT-FPS2) for qRT-PCR analysis were designed by Primer Premier 5.0 software and listed in **Supplementary Table S1**. The *β-actin* gene was used as en endogenous control to normalize expression. qRT-PCR were done with Roche Light Cycler 480. SYBR-green was used as instruction of manufacture. The PCR conditions were as follows: an initial incubation at 95°C for 3 min and then cycling at 95°C for 10 s, 60°C for 20 s and 72°C for 1 s for 40 cycles. There were five samples in each group and each sample was repeated for three times to insure the credibility of the data. The expression levels of *TwFPS1*&*2* were analyzed by the 2^-ΔΔCT^ (Livak and Schmittgen, 2001).

### Measurement of Celastrol Content

Cell suspensions were harvested as described by Su et al. (2014). 0.1 g cell suspensions were added with 1 ml 80% (v/v) methanol, soaked overnight at 4°C. Then the samples were extracted an ultrasonic water bath for 10 min. After centrifugation for 2 min, 10000 rpm at room temperature. The supernatant was extracted and concentrated by passing N_2_ at the opening of the tube. Stock solutions of the standards celastrol was accurately weighed, dissolved in methanol at 1.0 mg/mL, and diluted with methanol in suitable quantities to carry out a working solution used for calibration curve. Then all the samples were dissolved in 200 μl 80% (v/v) methanol and filtered through a 0.22 μm membrane filter before UPLC analysis.

The analyses were conducted using a ACQUITY UPLC I-Class system (Waters, United States) equipped with a PDA e» Detector, Sample Manager-FTN, Binary Solvent Manager. The chromatographic separation was conducted using an ACQUITY UPLC HSS T3 analytical column (1.8 μm, 2.1 × 100 mm) protected by a precolumn and kept at 40°C. The mobile phase consisting of a mixture of 0.05% (v/v) acetic acid in water (A) and 0.05% (v/v) acetic acid in acetonitrile (B) was set at a flow rate of 0.4 mL/min. The gradient program: 30% B at 0–5 min, 35% B at 5–15 min, 70% B at 15–21 min, 90% B at 21–21.5 min, 30% B at 21.5–24 min. The detection wavelength was 425 nm, and UV spectra from 190 to 500 nm were also recorded. The injection volume was 5 μL.

## Results

### Hygromycin Selection of Transformed Cells

From the **Supplementary Figure S1** shown on, the concentration was greater than 30 mg/L could brought out browning and death rapidly in cell suspensions. It could appear inhibition of growth, even death in cell suspensions with a concentration between 3 mg/L to 20 mg/L. However, it cannot inhibit the growth of cell suspensions effectively with a content of 0.1 mg/L to 2 mg/L. If that was used for the screening of resistance groups may cause a plenty of false positives. Therefore, the concentration 2.5 mg/L could inhibit the growth of suspension effectively and cannot cause cell death quickly.

### Range Analysis of Orthogonal Design

Using particle bombardment for cell suspensions transformation, seven parameters may impact the effect: target tissue, helium pressure, chamber vacuum pressure, number of bombardments, precipitation agents, plasmid DNA concentration and post-bombardment incubation time. In this research, the *egfp* were introduced by observing fluorescence spots under LSCM.

The range analysis was showed in **Table 3**, and it can be carried out a preliminary analysis that the order of parameters influence to bombardment effect was: post-bombardment incubation time > plasmid DNA concentration > chamber vacuum pressure > precipitation agents > target tissue > number of bombardment > helium pressure. This result could be noticed from the R’ ($R^{\prime }=dR\sqrt{r}$). In other words, post-bombardment incubation time, plasmid DNA concentration and chamber vacuum pressure were very significant whereas helium pressure was less important.

**Table 3:** Range analysis result of test L_18_ (6 × 3^6^).

Factor	Level 1	Level 2	Level 3	Level 4	Level 5	Level 6	R’
Target tissue distance (cm)	116.58	91.92	67.58	-	-	-	44.13
Helium pressure (psi)	77.17	108.17	90.75	-	-	-	27.92
Chamber vacuum pressure (mmHg)	60.58	98.75	116.75	-	-	-	50.59
Number of bombardments	85.00	75.50	115.58	-	-	-	36.10
Precipitation agents	114.17	58.75	103.17	-	-	-	49.91
Plasmid DNA (μg)	70.33	71.33	134.42	-	-	-	57.72
Time (hours)	103.50	162.67	86.50	23.67	47.17	128.67	125.98


**FIGURE 1:**
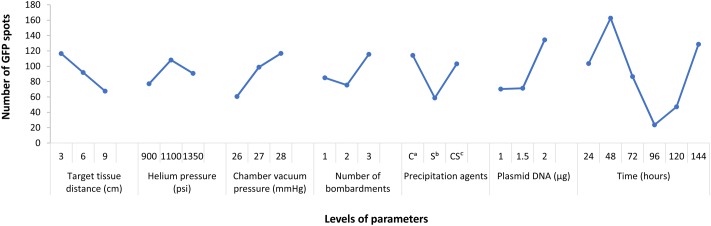
Parameters affecting *egfp* expression in *T. wilfordii* cell suspensions. Data are presented as mean ± SE (*n* = 6).

From the result of **Figure 1**, it indicated that the differences of several levels in each factor. Among them, the peak of the curve showed the significant level. Specifically, target tissue distance: 3 cm (level-1), helium pressure: 1100 psi (level-2), chamber vacuum pressure: 28 mmHg (level-3), number of bombardments: three times (level-3), precipitation agents: calcium chloride only (level-1), plasmid DNA: 2 μg (level-3), post-bombardment incubation time: 48 h (level-2). Thus, we knew which level we could choose in each factor. Thus, we knew which level we could choose in each factor.

### Variance Analysis of Orthogonal Design

In order to find out the significantly different from seven factors, variance analysis was performed, which was a necessary procedure to analyze the significance of various factors. **Table 4** showed the results of variance analysis. The probability values (*p*) showed that post-bombardment incubation time was highly significant (*p* < 0.01), number of bombardments and plasmid DNA were significant (*p* < 0.05).

**Table 4:** Variance analysis for the effect of control factors on the *egfp.*

Source	SS^a^	DF^b^	MS^c^	*F*^d^	*P*-value
Target tissue distance (cm)	6695.06	2	3347.53	1.19	0.328
Helium pressure (psi)	3266.89	2	1633.44	0.58	0.570
Chamber vacuum pressure (mmHg)	7419.39	2	3709.69	1.32	0.293
Number of bombardments	28924.39	2	14462.19	5.13	0.017*
Precipitation agents	6767.06	2	3383.53	1.20	0.324
Plasmid DNA (μg)	20213.56	2	10106.78	3.59	0.049*
Time (hours)	84467.56	5	16893.51	5.99	0.002**
Error	50729.00	18	2818.28		
Total	208482.89	35			


*^a^SS, Sums of Squares. ^b^DF, Degree of Freedom. ^c^MS, Adjusted Mean Sums of Squares. ^d^*F*-test statistic. ^∗^0.01 < *P* < 0.05: Number of bombardments, Plasmid DNA. ^∗∗^*P* < 0.01: Time.*

The effect of transformation was valuated with the expression of *egfp*. Post-bombardment incubation time, plasmid DNA concentration and number of bombardment were very significant whereas others were less important. Thus, the primary and secondary sequence of transformation by particle bombardment was: G, D, F, the optimal combination was: G2D3F3. Then combining range analysis with variance analysis, the comprehensive evaluation to determine the best transformation conditions was: A1B2C3D3E1F3G2; that was: target tissue distance 3 cm, helium pressure 1100 psi, chamber vacuum pressure 28 mmHg, number of bombardments three times, precipitation agents CaCl_2_ only, plasmid DNA concentration 2 μg, post-bombardment incubation time 48 h.

### Image Based Analysis of Transient Efficiency

**FIGURE 2:**
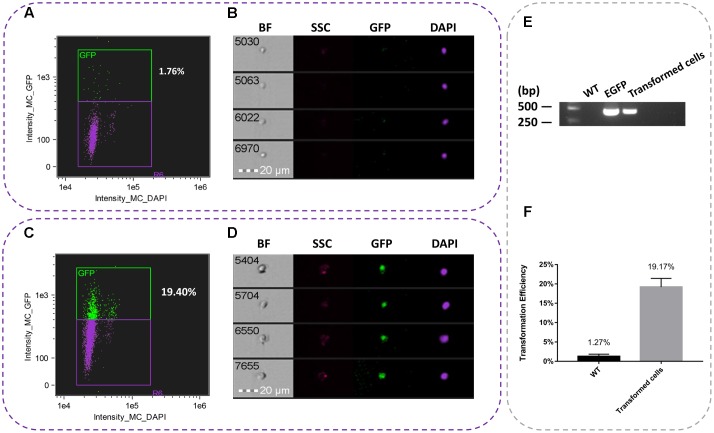
Representative pictures of single nuclei after transformation. **(A)** Percent of *egfp* in WT suspension cells. **(B)** The nuclei image of untransformed WT suspension cells. **(C)** Percent of *egfp* in transient cell suspensions. **(D)** The nuclei of transient suspension cells. **(E)** PCR analysis of transformed suspension cells from *T. wilfordii*. PCR fragments amplified from the pBI1300-EGFP primers. **(F)** Transformation efficiency of transient and WT cell suspensions.

On the basis of the combination of conditions, a total of 10,000 events were acquired and data were collected from three independent flow cytometric experiments. Using imaging flow cytometry analysis, we found that the average transformation efficiency was 19.17% (**Figure 2F**). As shown in **Figures 2A,B**, the nuclei of WT cell suspensions were only detected the DAPI fluorescence, while the nuclei of transformed cells were detected both GFP and DAPI fluorescence (**Figures 2C,D**). All the samples were performed for PCR analysis was accomplished using the pBI1300-EGFP F/R primers (**Supplementary Table S1**). It was showed the presence of 420 bp product consistent with the fragment *egfp* gene, reflecting the presence of the *egfp* transcript in the cell suspensions (**Figure 2E**).

### Identification of *TwFPS1*&*2* Transformed Cells

Transformation in *T. wilfordii* cell suspensions were achieved by particle bombardment. After 2 days following bombardment, all the cell suspensions were transferred to petri-dish containing MS medium supplemented with 0.5 mg/L 2,4-D, 0.1 mg/L KT, 0.5 mg/L IBA and 2.5 mg/L hygromycin. Two putative transformed cell lines were obtained after several rounds of selection (over 2 months) on MS described above.

**FIGURE 3:**
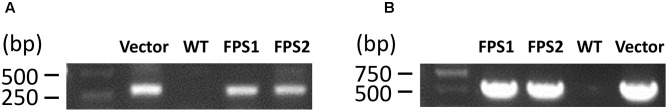
PCR analysis of the transformed cell suspensions T3 from *T. wilfordii*. **(A)** PCR fragments amplified using the pBI1300-EGFP primers. **(B)** PCR fragments amplified using the Hm primers.

These putative transformed cell lines were initially identified by PCR. Two pairs of primers (Hm-F/R; pBI1300-EGFP-F/R) were used to amplify part of the vector pH7WG2D sequence along with the FPS gene to verify the hygromycin gene and *egfp* gene. PCR analysis revealed 309 bp fragment and 512 bp fragment amplification of the hygromycin gene and *egfp* gene. The presence of PCR product in two transformed cell lines thus confirmed the integration of the hygromycin and *egfp* genes into the genome, while the WT cell suspensions did not show any PCR amplification (**Figure 3**).

**FIGURE 4:**
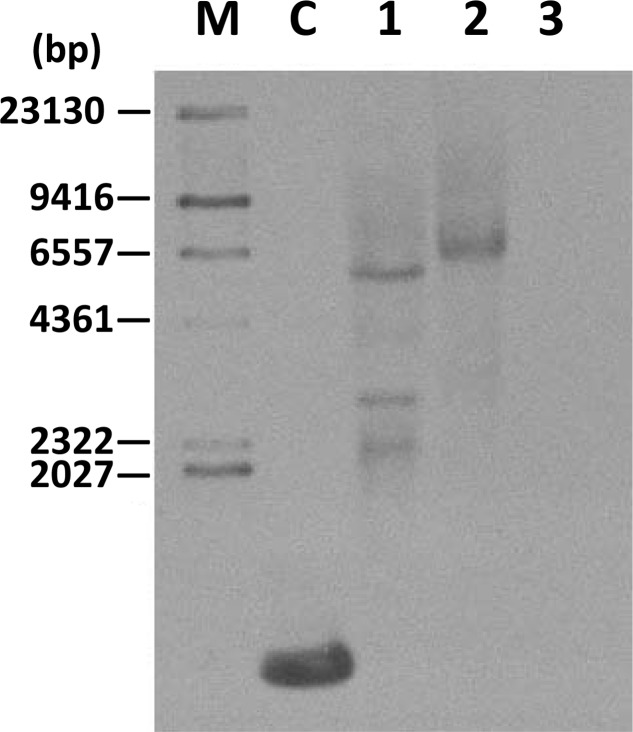
Southern blot analysis of two transformed cell lines. Total DNA (10 μg) was digested with *SacI*. Transformed T3 cell lines *TwFPS1* had three fragments and *TwFPS2* had one fragments, while no hybridization signal was detected in WT cell suspensions. Lane C: part of hygromycin gene fragment, Lane 1: *TwFPS1* T3 transgenic cell lines, Lane 2: *TwFPS2* T3 transgenic cell lines, Lane 3: WT cell suspensions.

Southern blot analysis was carried out using a hygromycin probe on two transgenic cell lines (*TwFPS1* and *TwFPS2*) to verify the integration of the hygromycin gene into the genome. Southern blot hybridization analysis shown in the **Figure 4**. There are three fragment in transgenic line *TwFPS1* and one fragment in transgenic line *TwFPS2*, while no hybridization signal in WT non-transformed cell suspensions was detected.

### Expression Analysis of *TwFPS1*&*2* from Transformed Cell Suspensions

**FIGURE 5:**
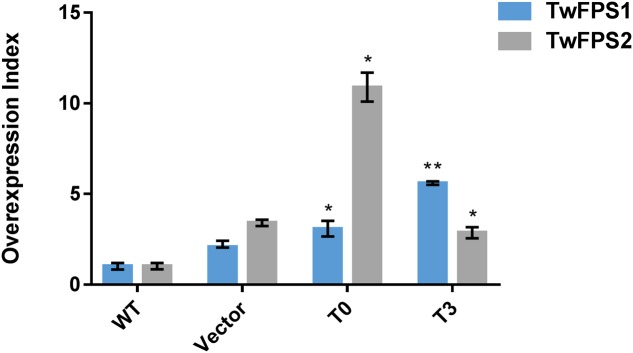
*TwFPS1*&*2* mRNA expression in cell suspensions T0 and T3 using real-time quantitative PCR. Expression in WT cell suspensions was set as 1. Data are presented as mean ± SE (*n* = 5). Asterisk represents statistical difference (^∗^*P* < 0.05, ^∗∗^*P* < 0.01).

Followed the method of combination A1B2C3D3E1F3G2, the transformation was performed again and the conversion efficiency was higher than before, as shown in **Figure 5**. To validate the efficiency of overexpression of *TwFPS1*&*2* from transformed *T. wilfordii* cell suspensions, the relative transcript level of *TwFPS1*&*2* gene was measured by real-time quantitative PCR. The two genes of different overexpression groups had alike pattern after transformation by particle bombardment. The relative expression of *TwFPS1* and *TwFPS2* in T0 cell suspensions were significantly increased (*P* < 0.05) approximately 3.06-fold and 10.87-fold higher, respectively, than in WT cell suspensions. The relative expression of *TwFPS1* and *TwFPS2* in T3 cell suspensions were significantly increased (*P* < 0.05) approximately 5.60-fold and 2.85-fold higher, respectively, than in WT cell suspensions. It indicated that the overexpression of *TwFPS1* and *TwFPS2* by particle bombardment could significantly enhanced the relative expression of the two genes in *T. wilfordii* cell suspensions.

### Content of Celastrol from Transformed Cells

In accordance with the method of combination A1B2C3D3E1F3G2, the transformation was accomplished again and the conversion efficiency was higher than before. The calibration curve was accomplished by plotting the corrected peak area (Y) for every standard against its concentration (X). According to the chromatographic conditions to determine the peak area, the calibration curve showed bright linear regression: *y* = 2678.4x - 261.29 (*R*^2^ = 0.9998) (**Supplementary Figure S2**).

**FIGURE 6:**
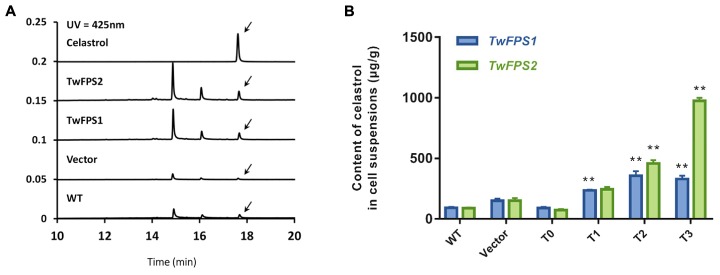
Content of celastrol in transformed *T. wilfordii* cell suspensions. **(A)** A UPLC analysis of the products in transformed T0 cell suspensions from *T. wilfordii*. The arrow represents the peak of celastrol. **(B)** Content of celastrol in transformed *T. wilfordii* T0 to T3 cell suspensions. WT cell suspensions was set as 1. Data are presented as mean ± SE (*n* = 5). The asterisk represents a significant difference (^∗∗^*P* < 0.01).

Furthermore, the content of celastrol in transformed cells was measured (**Figure 6A**). The UPLC retention time (RT) of celastrol in untransformed WT cells was 17.666 min; the retention time in empty vector transformed in cells was 17.625 min; the retention time in transformed cells overexpressing *TwFPS1* was 17.674 min; and the retention time in transformed cells overexpressing *TwFPS2* was 17.661 min.

The content of celastrol in WT cell suspensions was 91.73 ± 6.27 μg/g, in vector pH7WG2D cell suspensions was 148.95 ± 14.69 μg/g. The accumulation of celastrol in transgenic *T. wilfordii* cells T0 transformed with *TwFPS1* was 90.95 ± 6.74 μg/g, transformed with *TwFPS2* was 73.70 ± 5.27 μg/g. The accumulation of celastrol in transgenic *T. wilfordii* cell suspensions T1 transformed with *TwFPS1* was 235.77 ± 5.11 μg/g, transformed with *TwFPS2* was 244.28.08 ± 16.23 μg/g. The accumulation of celastrol in transgenic *T. wilfordii* cell suspensions T2 transformed with *TwFPS1* was 355.34 ± 32.29 μg/g, transformed with *TwFPS2* was 456.84 ± 23.87 μg/g. After transformation of *TwFPS1* and *TwFPS2*, celastrol in cell suspensions T3 was increased significantly (*P* < 0.01), about 3.48 fold and 10.60 fold higher than in WT cell suspensions (**Figure 6B**). It indicated that transformation and overexpression of *TwFPS1* and *TwFPS2* by particle bombardment could significantly enhanced the content of celastrol in *T. wilfordii* cell suspensions.

## Discussion

Particle bombardment has been widely used to transform exogenous genes into plant tissues and has a major impact on basic plant biotechnology (Ahmad et al., 2017). The present work was to optimize different parameters in particle bombardment-mediated plant transformation that could enhance stable integration of the target genes in *T. wilfordii* cell suspensions. The average efficiency of transient transformation was 19.17% and higher than those reported previously (Song et al., 2006).

Orthogonal experiment was taken part instead of control variate method. Selected representative sites for test through the typical experimental results of the analysis, understand the comprehensive test, in order to realize the optimization of process. In 1951, the Japanese statisticians Taguchi (Taguchi, 1960) proposed an optimization orthogonal table rule according to the test. Orthogonal table become a basic tool of orthogonal test design and it make the orthogonal experiment with the dispersion and tidy comparability. This cannot only determine the effect of primary and secondary order of the factors according to the orthogonal table, but also be analyzed the influence degree of various factors on the index. Then obtain the optimal combination and find out the optimal conditions. In this paper optimization was studied by an orthogonal Taguchi array of L_18_ type. The results showed the main influence factors: post-bombardment incubation time, number of bombardments and plasmid DNA concentration; transform conditions: target tissue distance 3 cm, helium pressure 1100 psi, chamber vacuum pressure 28 mmHg, number of bombardments three times, precipitation agents CaCl_2_ only, plasmid DNA concentration 2 μg, post-bombardment incubation time 48 h.

The helium pressure had significant effect on particle bombardment-mediated transformation. Low pressures could reduce transient GFP expression, as plasmid was not able to reach recipient tissues or cells. While higher pressure may cause injury of plant tissues or cells. We found that helium pressure of 1100 psi has the highest efficiency of transformation in *T. wilfordii* cell suspensions. Likewise, this level of helium pressure has been reported in transgenic *Hypericum perforatum* plants (Franklin et al., 2007).

The target tissue distance of 3 cm was an important factor in improving stable transformation. It was different from previous researches that on *Catharanthus roseus* (Guirimand et al., 2009) 6 cm distance was reported to be optimal. The distance from the macrocarrier to target tissue can also affect the velocity of microparticles and consequently transformation rates (Petrillo et al., 2008). This distance could not only ensure the distribution of DNA microcarrier over the target tissue, but also largely reduce the damage (Vasudevan et al., 2013). More than over, tissue dislocation and mechanical damage was not observed at too short microcarrier travel distance in our work. This might be related to *T. wilfordii* cellular morphology, which needs more research. Contrary to this, 9 cm microprojectile travel distance was reported to be optimum for banana (Mahdavi et al., 2014), wheat (Gharanjik et al., 2008) and cumin embroys (Singh et al., 2010).

Triple bombardment resulted the highest efficiency of transformation that was different from previous report (Jagga-Chugh et al., 2012). In the present work, triple bombardment per plate of cell suspension, was found to be optimal in GFP expression and transformation efficiency. That has been shown in *Dendrobium orchid* (Kuehnle and Sugii, 1992) and *Carica papaya* (Fitch et al., 1990). *C. papaya* belongs to wooden herb, same as *T. wilfordii.* It showed that the bombardment times might relate to the degree of lignification. Our result showed no significant difference in GFP expression with single and multiple bombardments on *T. wilfordii* cell suspensions. This was reported on wheat tissues (Rascogaunt et al., 1999). Moreover, it could not cause the tissue damage with higher helium pressures.

Here we have to take into consideration that *T. wilfordii* is woody liana and its explant grow slowly, we need to develop a rapid way of transformation. Osmotic treatment can decrease the turgor pressure in cells and avoid cell rupture caused by particle bombardment (Terada et al., 2010). Meanwhile it can increase the survival rate (Egertsdotter and Clapham, 2013). We choose mannitol and sorbitol as penetrant resulted in growth retardation of suspension cells in pre-experiment. However, the growth of the receptor material condition is the key factor of transformation of particle bombardment. No matter what the explant as the object, the receptor material must be healthily growing, otherwise it will affect the physiological status of donor tissue and the totipotency of cell transforming (Bhalla, 2006). Then we just conduct regular treatment of preincubate.

In based on tissue culture of *T. wilfordii* genetic transformation system, the antagonism of cells screening is an important link. There is not a clear standard that how to determine concentration of screening reagents mass so far. In practice using commonly of screening reagent dosage, which can inhibit the growth of the transformed cells effectively and transformation cells grow without limits. The toxicity mechanism of hygromycin is to interfere with ribosomes to bind to elongation factor EF-2 in plant cells chloroplast and mitochondria and can inhibit the prolongation of peptide chain. This study identified accordingly the critical concentration of hygromycin selection was 2.5 mg/L, that was used for future positive selection steps.

Under this transformation system, the results of overexpression elucidated that *TwFPS1*&*2* was highly expressed in transgenic cells with vector pH7WG2D and metabolite detection showed that celastrol concentration was highly increased as well. That was to say, this transformation system was efficient for gene transformation and it could be used for gene function characterization. In this paper, we got the same conclusion. Triterpenoid celastrol was 10-fold increase compared with that in WT cell suspensions. It suggested that overexpression FPS can change the biosynthesis of triterpenoid. Used expression to improve the transcription level of FPS was beneficial to strengthen metabolism flow turn to triterpenoid synthesis and promote accumulation of celastrol.

## Author Contributions

YuZ and WG wrote the manuscript. YuZ, YiZ, PS, and JY performed the experiments. WG and LH supervised the research.

## Conflict of Interest Statement

The authors declare that the research was conducted in the absence of any commercial or financial relationships that could be construed as a potential conflict of interest.

## Supplementary Material

**FIGURE S1 |** Effects of hygromycin in *T. wilfordii* cell suspensions.

**FIGURE S2 |** The calibration curve of standard celastrol accomplished by plotting the corrected peak area (*Y*) for every standard against its concentration (*X*).

**FIGURE S3 |** The map of plasmid PBI1300-EGFP.

**FIGURE S4 |** The map of plasmid pENTR/SD/D-TOPO.

**FIGURE S5 |** The map of plasmid pH7WG2D.

**TABLE S1 |** Primers for all PCR.

**TABLE S2 |** Range analysis of the orthogonal experiment and its specific calculation.

**TABLE S3 |** Variance analysis of the orthogonal experiment and its specific calculation.
