# Supplementary figures and images for: Genetic Transformation System for Woody Plant Tripterygium wilfordii and Its Application to Product Natural Celastrol
Source: Front Plant Sci. 2018 Jan 9;8:2221. doi: 10.3389/fpls.2017.02221 (PMC5767223; doi:10.3389/fpls.2017.02221)

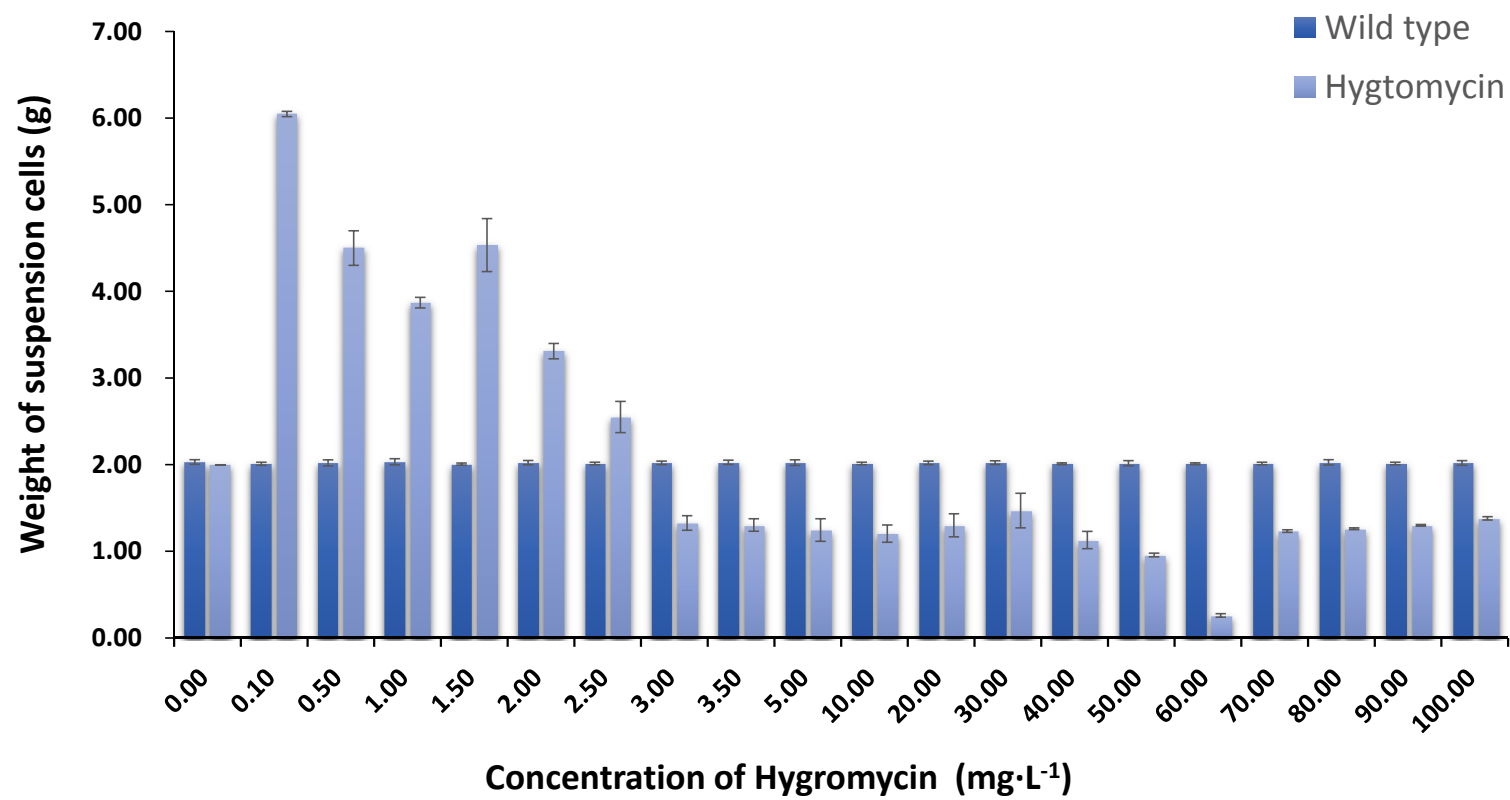

Supplement: Supplementary file 1 [file Image_1.PDF]

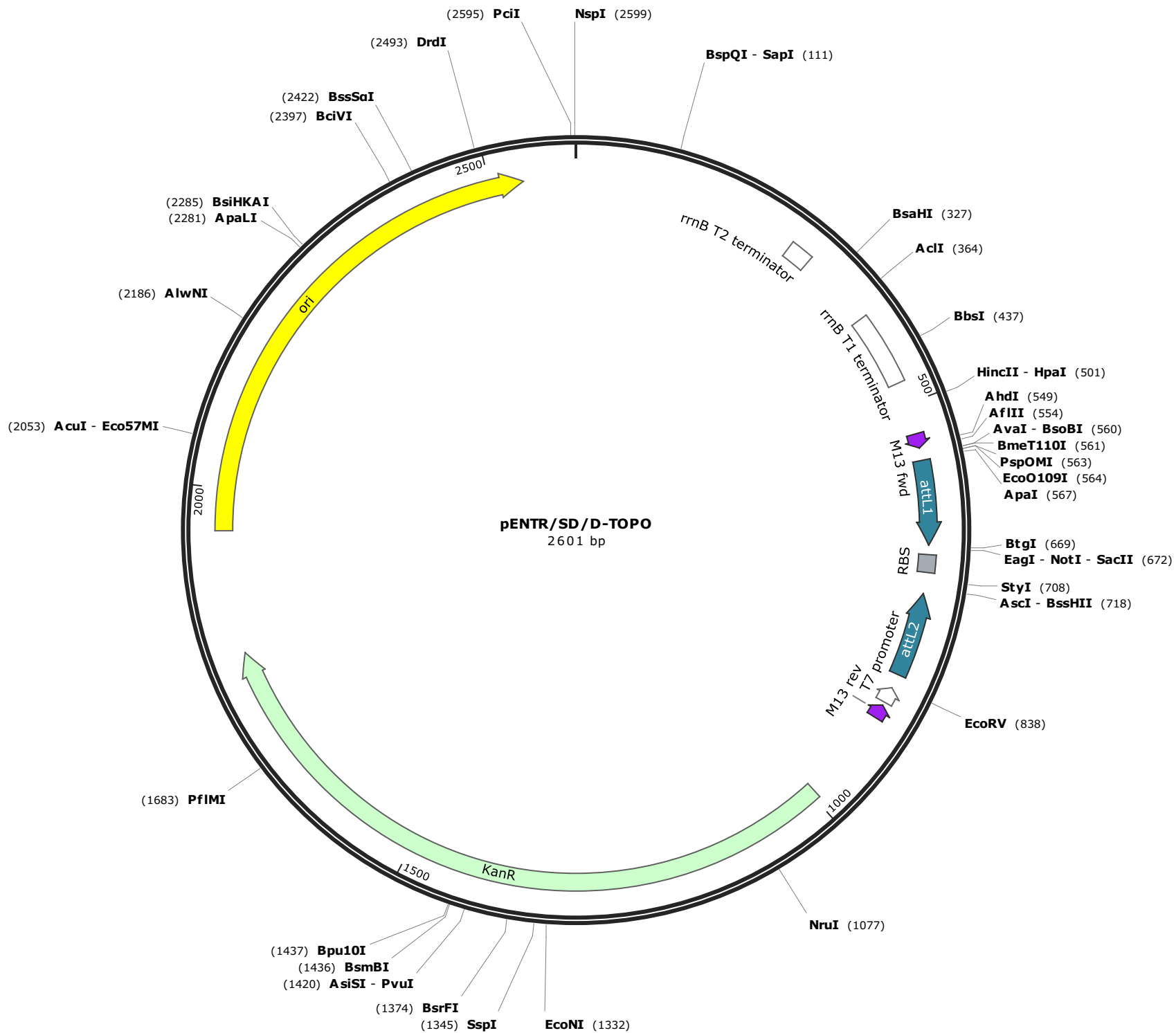

Supplement: Supplementary file 4 [file Image_4.PDF]

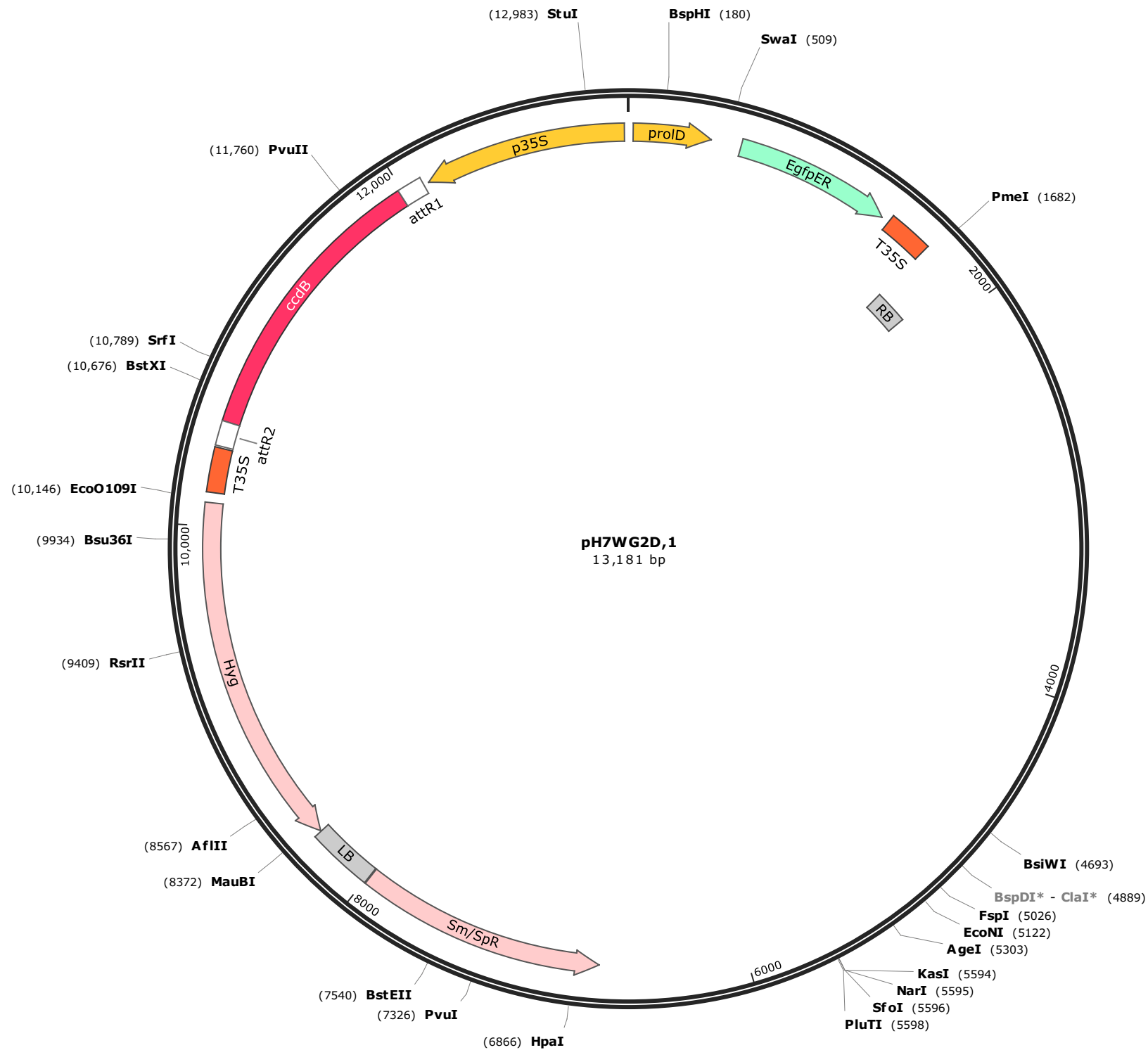

Supplement: Supplementary file 5 [file Image_5.PDF]
