# Supplementary material for: Genetic Transformation System for Woody Plant Tripterygium wilfordii and Its Application to Product Natural Celastrol
Source: Front Plant Sci. 2018 Jan 9;8:2221. doi: 10.3389/fpls.2017.02221 (PMC5767223; doi:10.3389/fpls.2017.02221)
Supplement: Supplementary file 2 [file Image_2.PDF]

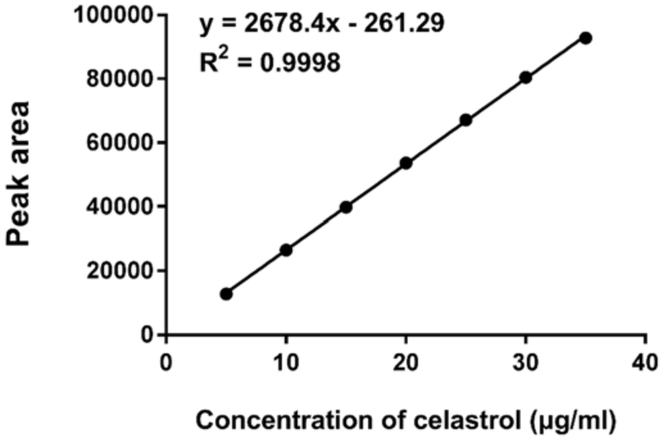

Fig. S2 The calibration curve of standard celastrol accomplished by plotting the corrected peak area (Y) for every standard against its concentration (X).
