# Supplementary material for: Genetic Transformation System for Woody Plant Tripterygium wilfordii and Its Application to Product Natural Celastrol
Source: Front Plant Sci. 2018 Jan 9;8:2221. doi: 10.3389/fpls.2017.02221 (PMC5767223; doi:10.3389/fpls.2017.02221)
Supplement: Supplementary file 6 [file Table_1.PDF]

Supplemental Table 1 Primers for all PCR

Gene	Forward primer	Reverse primer
FPS1-att	CACCATGAGCGACACCAAGTCCAAGT	CTACTTCTCTCGCTTGTATATT
FPS2-att	CACCATGGCGGATCTCAAGTCAACGT	CTACTTCTGTCTCTTGTATATC
PH7-F	TCATTTGGAGAGGACTCCGG	
PBI1300-EGFP	GTAAACGGCCACAAGTTCAGCG	GATGCCGTTCTTCTGCTTGTCG
PH7-EGFP	ACCCTCGTGACCACCCTGAC	AGTTCACCTTGATGCCGTTCTT
Hm	CGTTATGTTTATCGGCACT	TTGGCGACCTCGTATTGG
qRT-β-Actin	AGGAACCACCGATCCAGACA	GGTGCCCTGAGGTCCTGTT
qRT-FPS1	GGGTGTATTTGCGGAGT	CGGCAGAATCTAATGGAG
qRT-FPS2	CAGACCCTCACCTTCCATT	AAGAGTAACCATAAGCAGCAGAC
