# Supplementary material for: Genetic Transformation System for Woody Plant Tripterygium wilfordii and Its Application to Product Natural Celastrol
Source: Front Plant Sci. 2018 Jan 9;8:2221. doi: 10.3389/fpls.2017.02221 (PMC5767223; doi:10.3389/fpls.2017.02221)
Supplement: Supplementary file 3 [file Image_3.PDF]

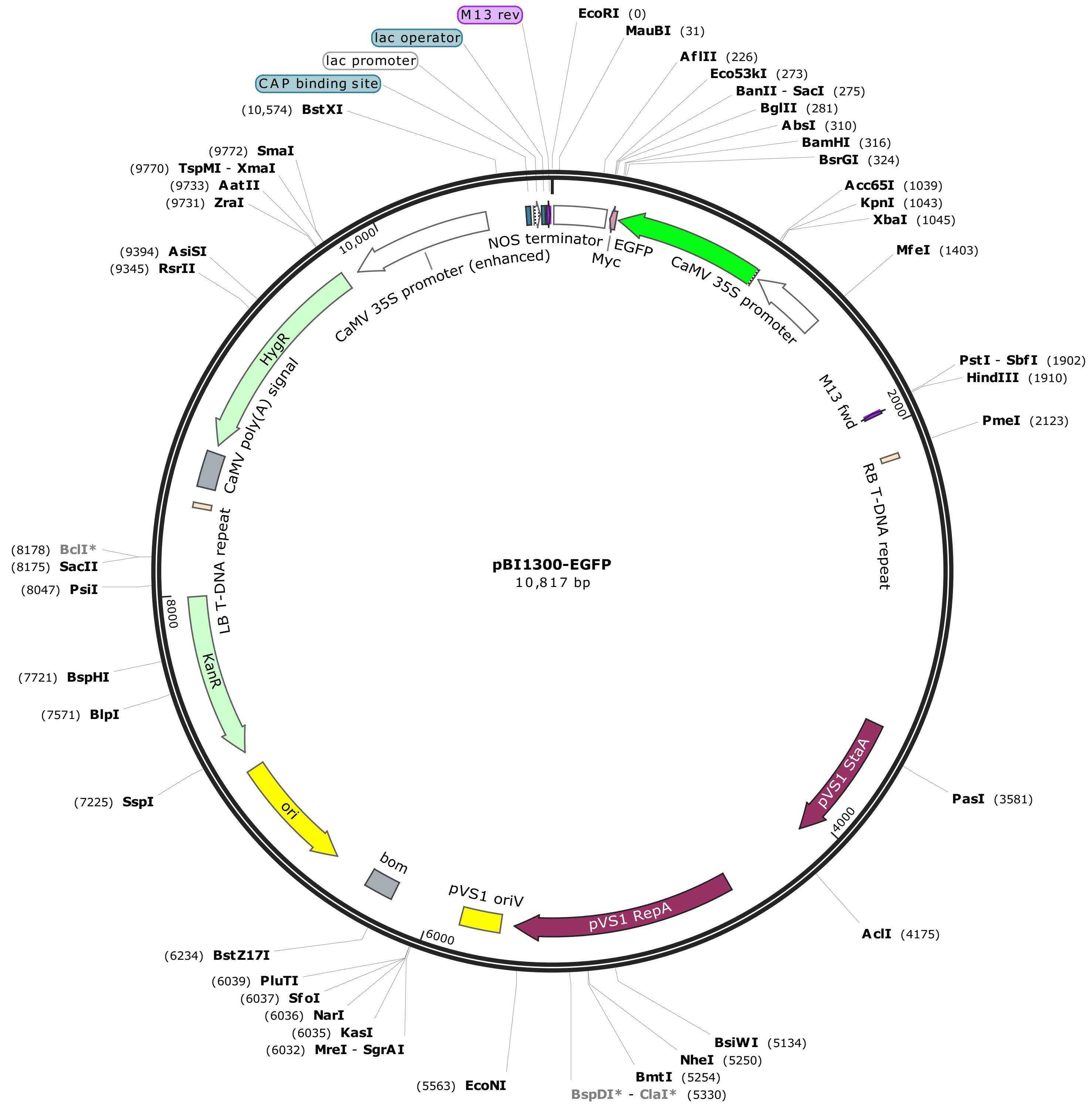
Created with SnapGene®
pBI1300-EGFP
10,817 bp
M13 rev
lac operator
lac promoter
CAP binding site
(10,574) BstXI
EcoRI (0)
MauBI (31)
AflII (226)
Eco53kI (273)
BanII - SacI (275)
BglII (281)
AbsI (310)
BamHI (316)
BsrGI (324)
Acc65I (1039)
KpnI (1043)
XbaI (1045)
MfeI (1403)
PstI - SbfI (1902)
HindIII (1910)
PmeI (2123)
PasI (3581)
AclI (4175)
BsiWI (5134)
NheI (5250)
BmtI (5254)
BspDI* - ClaI* (5330)
(5563) EcoNI
(6032) MreI - SgrAI
(6035) KasI
(6036) NarI
(6037) SfoI
(6039) PluTI
(6234) BstZ17I
(7225) SspI
(7571) BlpI
(7721) BspHI
(8047) PsiI
(8175) SacII
(8178) BclI*
(9345) RsrII
(9394) AsiSI
(9731) ZraI
(9733) AatII
(9770) TspMI - XmaI
(9772) SmaI
NOS terminator
EGFP
Myc
CaMV 35S promoter
CaMV 35S promoter (enhanced)
M13 fwd
RB T-DNA repeat
pVS1 StaA
pVS1 RepA
pVS1 oriV
bom
ori
KanR
LB T-DNA repeat
CaMV poly(A) signal
HygR
10,000
2000
4000
6000
8000
